# Supplementary material for: Calorie restriction prevents age-related changes in the intestinal microbiota
Source: Aging (Albany NY). 2021 Mar 10;13(5):6298–329. doi: 10.18632/aging.202753 (PMC7993711; doi:10.18632/aging.202753)
Supplement: Supplementary Figures [file aging-13-202753-s001.pdf]

SUPPLEMENTARY FIGURES

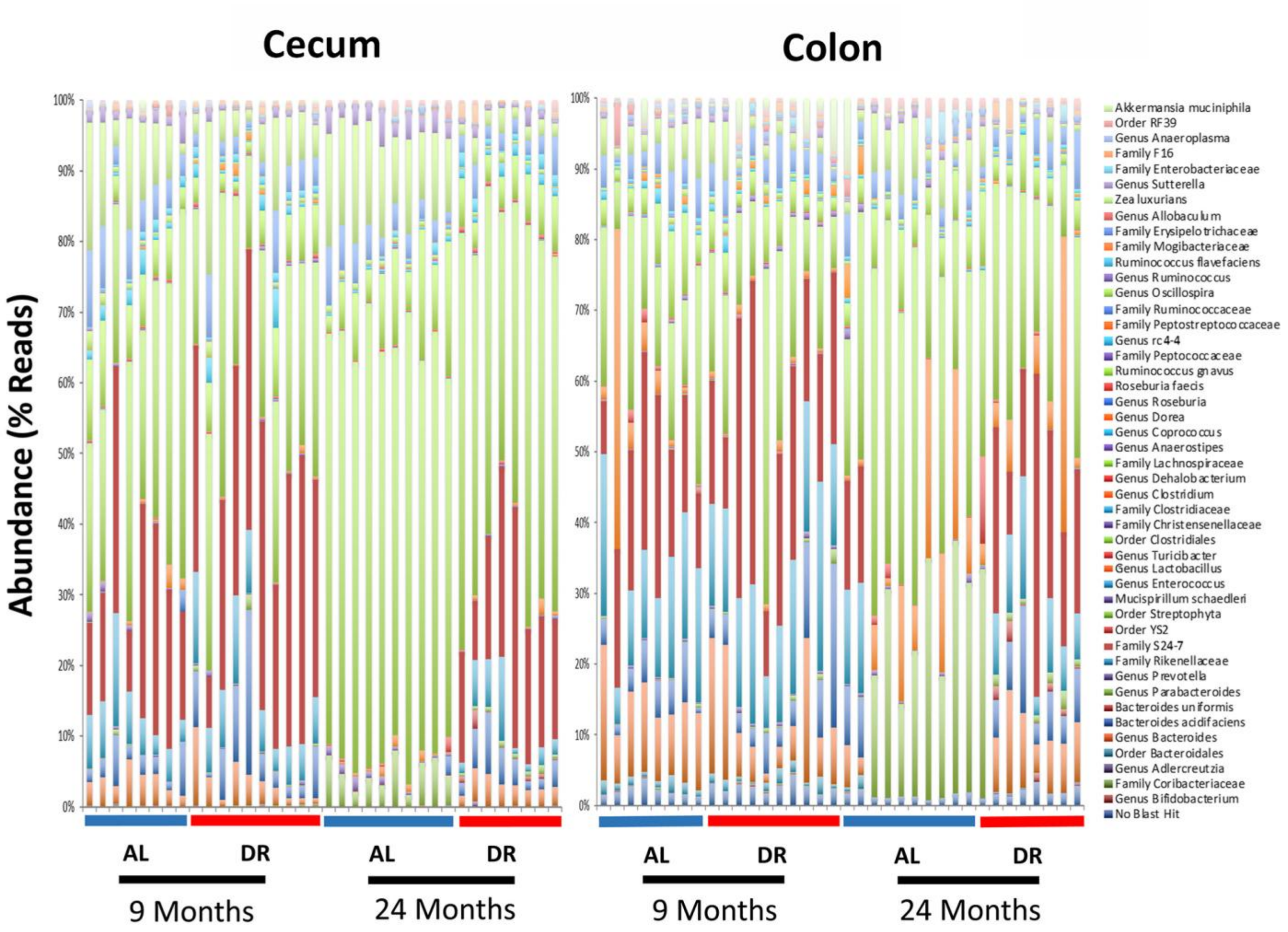

Supplementary Figure 1. Relative abundance of all the microbes in the cecum and colon of C57BL/6J mice.

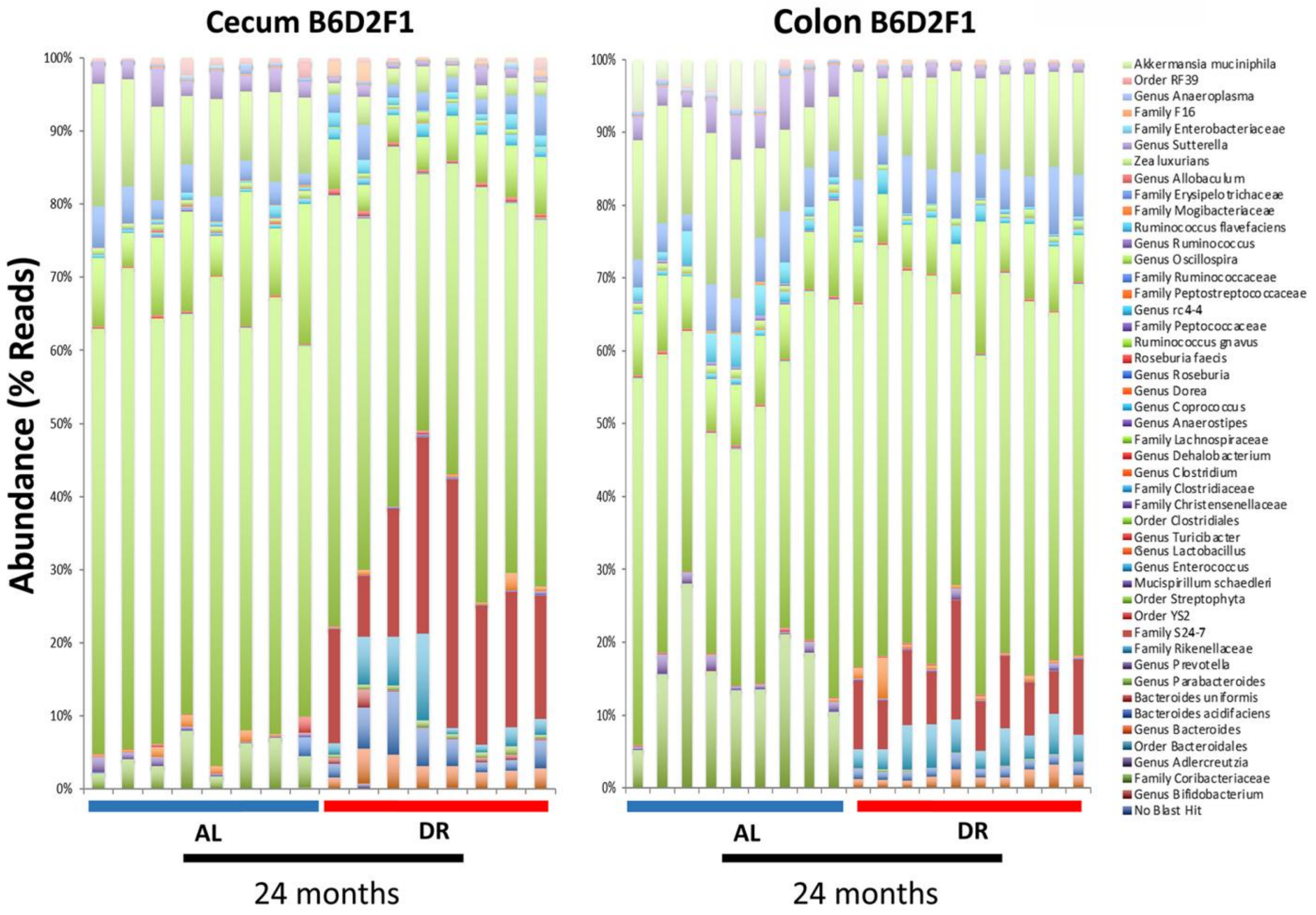

Supplementary Figure 2. **Relative abundance of all the microbes in the cecum and colon of the B6D2F1 mice.**

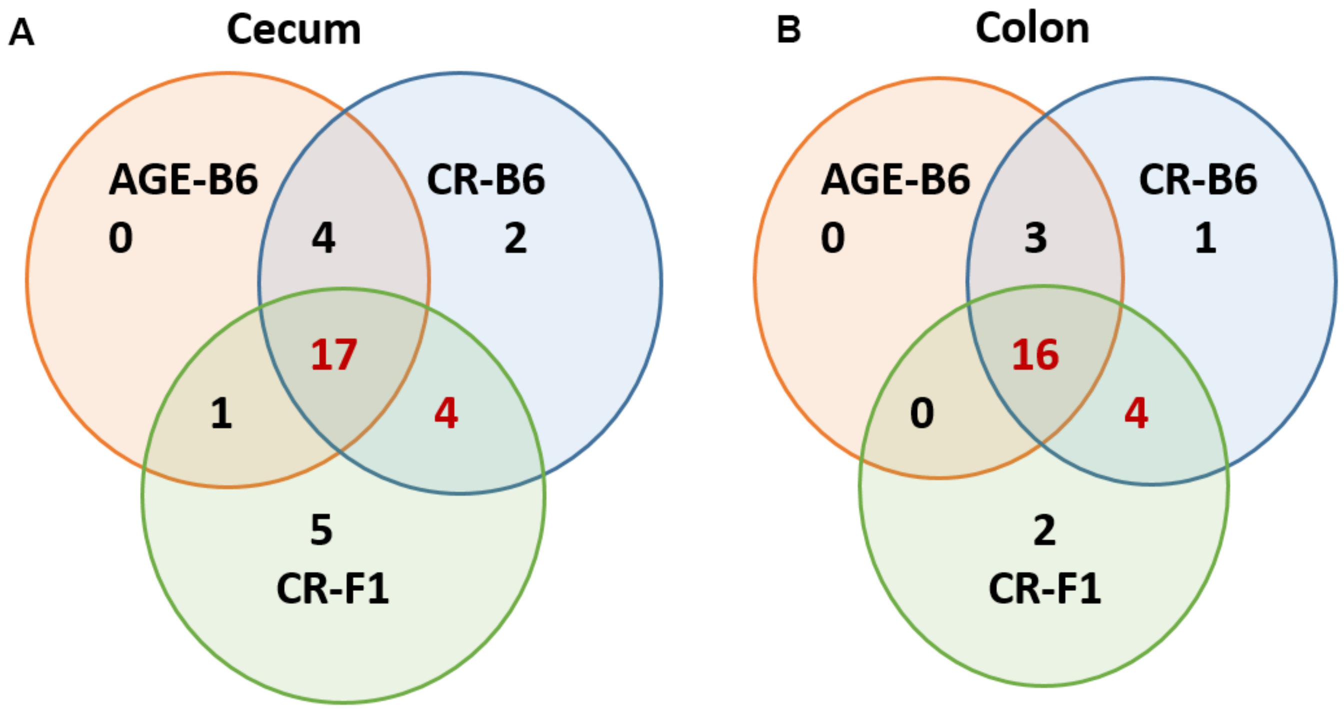

Supplementary Figure 3. Distribution of microbes that changed with CR in (**A**) Cecum and (**B**) Colon of both C57BL/6JN and B6D2F1 mice.
