# Supplementary material for: Calorie restriction prevents age-related changes in the intestinal microbiota
Source: Aging (Albany NY). 2021 Mar 10;13(5):6298–329. doi: 10.18632/aging.202753 (PMC7993711; doi:10.18632/aging.202753)
Supplement: Supplementary Tables 1, 2, 3 and 4 [file aging-13-202753-s002.pdf]

SUPPLEMENTARY TABLES

Supplemenatry Table 1. Microbiota composition of the cecum from adult and old C57BL/6 mice fed *ad libitum* (AL) or caloric restriction (CR).

	Adult AL	Adult CR	Old AL	Old CR
**Species**				
Akkermansia muciniphila	0.43 ± 0.36	3.25 ± 1.16[a]	0.0 ± 0.0	0.01 ± 0.01[d]
Bacteroides acidifaciens	4.33 ± 0.72	8.1 ± 2.08	0.0 ± 0.0[b]	7.15 ± 1.56[c]
Bacteroides uniformis	0.01 ± 0.0	0.0 ± 0.0	0.0 ± 0.0[b]	1.12 ± 0.39[cd]
Eubacterium cylindroides	0.01 ± 0.01	0.55 ± 0.16[a]	0.0 ± 0.0	0.0 ± 0.0[d]
Mucispirillum schaedleri	0.16 ± 0.02	0.11 ± 0.03	0.45 ± 0.13[b]	0.04 ± 0.01[c]
Ruminococcus gnavus	0.7 ± 0.12	0.49 ± 0.11	0.45 ± 0.04	0.26 ± 0.05[c]
**Genus**				
Allobaculum	0.88 ± 0.61	0.55 ± 0.25	0.0 ± 0.0	0.0 ± 0.0
Anaeroplasma	0.52 ± 0.16	0.0 ± 0.0[a]	0.04 ± 0.02[b]	0.0 ± 0.0
Bacteroides	13.23 ± 1.51	6.47 ± 1.74[a]	0.0 ± 0.0[b]	8.75 ± 1.26[c]
Coprococcus	1.13 ± 0.24	0.41 ± 0.05[a]	0.37 ± 0.06[b]	0.55 ± 0.12
Dehalobacterium	0.19 ± 0.03	0.17 ± 0.03	0.11 ± 0.02[b]	0.13 ± 0.03
Dorea	0.38 ± 0.16	1.66 ± 0.52[a]	0.05 ± 0.01	0.14 ± 0.04[cd]
Lactobacillus	6.26 ± 4.47	0.9 ± 0.1	12.46 ± 3.24	9.27 ± 5.64
Oscillospira	5.14 ± 0.75	1.61 ± 0.22[a]	6.36 ± 0.95	1.08 ± 0.41[c]
Parabacteroides	0.2 ± 0.05	0.56 ± 0.19	25.99 ± 2.89[b]	1.45 ± 0.22[cd]
Prevotella	0.26 ± 0.05	0.55 ± 0.09[a]	0.0 ± 0.0[b]	0.0 ± 0.0[d]
Ruminococcus	0.71 ± 0.13	0.37 ± 0.1[a]	1.07 ± 0.13	0.36 ± 0.15[c]
Turicibacter	0.52 ± 0.25	0.0 ± 0.0	1.85 ± 1.36	0.17 ± 0.1
rc4-4	0.47 ± 0.07	0.45 ± 0.05	0.29 ± 0.11	0.43 ± 0.08
**Family**				
Christensenellaceae	0.2 ± 0.04	0.79 ± 0.09[a]	0.04 ± 0.01[b]	0.08 ± 0.02[d]
Clostridiaceae	0.13 ± 0.03	0.09 ± 0.01	0.13 ± 0.06	0.1 ± 0.03
Enterobacteriaceae	0.04 ± 0.02	0.04 ± 0.02	0.91 ± 0.48	0.43 ± 0.3
Erysipelotrichaceae	0.47 ± 0.11	0.33 ± 0.12	0.98 ± 0.28	0.43 ± 0.08
F16	0.26 ± 0.06	0.61 ± 0.1[a]	0.0 ± 0.0[b]	1.17 ± 0.55[c]
Lachnospiraceae	6.05 ± 1.18	3.78 ± 0.63	6.72 ± 0.99	4.0 ± 0.78
Mogibacteriaceae	0.1 ± 0.01	0.09 ± 0.01	0.35 ± 0.08[b]	0.09 ± 0.02[c]
Rikenellaceae	14.93 ± 1.61	16.81 ± 1.66	0.0 ± 0.0[b]	9.2 ± 1.79[cd]
Ruminococcaceae	4.01 ± 0.51	3.9 ± 0.66	2.62 ± 0.29[b]	3.91 ± 0.92
S24-7	17.83 ± 2.37	24.17 ± 3.53	0.0 ± 0.0[b]	23.1 ± 4.78[c]
**Order**				
Order Bacteroidales	1.42 ± 0.2	1.21 ± 0.27	0.0 ± 0.0[b]	0.12 ± 0.03[cd]
Order Clostridiales	18.41 ± 2.37	20.93 ± 4.17	37.09 ± 4.01[b]	25.8 ± 3.16
Order RF32	0.16 ± 0.05	0.28 ± 0.12	0.0 ± 0.0[b]	0.0 ± 0.0
Order RF39	0.24 ± 0.06	0.55 ± 0.11[a]	1.67 ± 0.21[b]	0.67 ± 0.22[c]
Order YS2	0.22 ± 0.1	0.23 ± 0.07	0.0 ± 0.0	0.0 ± 0.0[d]

Each value represents the mean ± SEM of data generated from 8 to 10 mice per group. Significant differences between groups are shown for an FDR <0.05: a = Significant difference between adult AL and young CR; b = Significant difference between adult AL and old AL; c = Significant difference between old AL and old CR; d = Significant difference between adult CR and old CR.

Supplemenatry Table 2. Microbiota composition of colon from adult and old C57BL/6 mice fed *ad libitum* (AL) or caloric restriction (CR).

	Adult AL	Adult CR	Old AL	Old CR
**Species**				
Bacteroides acidifaciens	2.75 ± 0.84	6.71 ± 2.15	0.0 ± 0.0[b]	3.97 ± 0.89[c]
Bacteroides uniformis	0.0 ± 0.0	0.0 ± 0.0	0.0 ± 0.0	0.67 ± 0.29[cd]
Mucispirillum schaedleri	0.47 ± 0.18	0.19 ± 0.09	0.87 ± 0.2	0.24 ± 0.07[c]
Ruminococcus gnavus	0.79 ± 0.27	0.65 ± 0.17	0.78 ± 0.06	0.51 ± 0.13
**Genus**				
Anaeroplasma	0.37 ± 0.18	0.1 ± 0.08	0.01 ± 0.0	0.0 ± 0.0
Bacteroides	2.95 ± 0.67	3.54 ± 1.03	0.0 ± 0.0[b]	3.02 ± 0.41[c]
Coprococcus	1.28 ± 0.37	1.72 ± 0.54	0.44 ± 0.04[b]	1.34 ± 0.14[c]
Dehalobacterium	0.22 ± 0.05	0.39 ± 0.05[a]	0.24 ± 0.03	0.54 ± 0.06[c]
Dorea	0.08 ± 0.04	0.56 ± 0.17[a]	0.02 ± 0.01	0.07 ± 0.02[cd]
Lactobacillus	0.65 ± 0.34	0.26 ± 0.1	0.7 ± 0.2	0.57 ± 0.27
Oscillospira	8.18 ± 2.13	7.14 ± 1.91	13.13 ± 0.83[b]	2.36 ± 0.31[cd]
Parabacteroides	0.13 ± 0.08	0.41 ± 0.21	4.79 ± 0.7[b]	0.73 ± 0.08[c]
Ruminococcus	1.49 ± 0.41	0.79 ± 0.17	3.02 ± 0.34[b]	1.06 ± 0.29[c]
Turicibacter	0.04 ± 0.03	0.01 ± 0.0	0.38 ± 0.22	0.09 ± 0.06
rc4-4	0.52 ± 0.19	0.97 ± 0.12	0.44 ± 0.16	1.3 ± 0.24[c]
**Family**				
Christensenellaceae	0.11 ± 0.03	0.25 ± 0.05[a]	0.01 ± 0.0[b]	0.09 ± 0.02[cd]
Erysipelotrichaceae	0.08 ± 0.04	0.21 ± 0.09	0.3 ± 0.1	0.21 ± 0.05
F16	0.25 ± 0.08	0.48 ± 0.08	0.0 ± 0.0[b]	0.85 ± 0.34[c]
Lachnospiraceae	5.8 ± 1.35	5.15 ± 0.9	10.04 ± 1.84	5.82 ± 0.58
Rikenellaceae	5.52 ± 1.51	7.57 ± 1.02	0.01 ± 0.0[b]	4.17 ± 1.37[c]
Ruminococcaceae	5.74 ± 1.16	3.96 ± 0.9	4.28 ± 0.64	3.0 ± 0.5
S24-7	26.57 ± 4.76	31.62 ± 3.34	0.02 ± 0.01[b]	19.72 ± 2.75[cd]
Sphingobacteriaceae	0.32 ± 0.31	0.02 ± 0.0	0.29 ± 0.27	0.02 ± 0.0
**Order**				
Order Clostridiales	35.52 ± 3.77	27.0 ± 2.59	59.33 ± 1.49[b]	49.17 ± 2.67[cd]
Order RF39	0.16 ± 0.05	0.31 ± 0.07	0.89 ± 0.18[b]	0.48 ± 0.18

Each value represents the mean ± SEM of data generated from 8 to 10 mice per group. Significant differences between groups are shown for an FDR <0.05: a = significant difference between adult AL and adult CR; b = significant difference between adult AL and old AL; c = Significant difference between old AL and old CR; d = Significant difference between adult CR and old CR.

Supplemenatry Table 3. Microbiota composition of cecum and colon from old B6D2F1 mice fed ad libitum (AL) or caloric restriction (CR).

	Cecum		Colon	
	**Old AL**	**Old CR**	**Old AL**	**Old CR**
**Species**				
Akkermansia muciniphila	7.11 ± 2.01	0.0 ± 0.0a	3.06 ± 0.94	0.0 ± 0.0[a]
Bacteroides acidifaciens	0.0 ± 0.0	5.78 ± 0.43[a]	0.67 ± 0.67	1.4 ± 0.1
Mucispirillum schaedleri	0.2 ± 0.13	0.03 ± 0.01	1.1 ± 0.28	0.47 ± 0.15
Ruminococcus gnavus	0.52 ± 0.1	0.38 ± 0.06	0.79 ± 0.11	0.75 ± 0.06
**Genus**				
Adlercreutzia	0.09 ± 0.02	0.17 ± 0.02[a]	ND	ND
Anaeroplasma	0.17 ± 0.08	0.09 ± 0.09	ND	ND
Anaerostipes	0.26 ± 0.03	0.0 ± 0.0[a]	ND	ND
Bacteroides	0.0 ± 0.0	4.67 ± 0.63[a]	0.0 ± 0.0	1.67 ± 0.24[a]
Coprococcus	0.45 ± 0.07	0.75 ± 0.14	0.74 ± 0.14	1.29 ± 0.32
Dehalobacterium	0.26 ± 0.04	0.17 ± 0.01[a]	0.28 ± 0.04	0.27 ± 0.02
Lactobacillus	0.1 ± 0.04	5.09 ± 0.67[a]	0.06 ± 0.04	1.05 ± 0.52
Oscillospira	6.78 ± 0.36	5.16 ± 0.61[a]	12.68 ± 1.97	12.47 ± 0.68
Parabacteroides	30.72 ± 2.64	0.21 ± 0.05[a]	14.18 ± 2.51	0.11 ± 0.03[a]
Prevotella	0.0 ± 0.0	0.51 ± 0.14[a]	ND	ND
Ruminococcus	2.33 ± 0.38	0.56 ± 0.07[a]	3.97 ± 0.66	1.46 ± 0.1[a]
Turicibacter	0.6 ± 0.21	1.17 ± 0.18	0.18 ± 0.04	0.14 ± 0.02
rc4-4	5.01 ± 1.09	0.8 ± 0.15[a]	2.54 ± 0.55	0.32 ± 0.06[a]
**Family**				
Erysipelotrichaceae	0.92 ± 0.18	0.06 ± 0.02[a]	0.45 ± 0.09	0.04 ± 0.01[a]
F16	0.0 ± 0.0	1.05 ± 0.22[a]	0.0 ± 0.0	0.27 ± 0.03[a]
Lachnospiraceae	6.92 ± 0.67	3.83 ± 0.49[a]	8.64 ± 0.62	8.55 ± 1.15
Mogibacteriaceae	0.18 ± 0.02	0.09 ± 0.01[a]	0.16 ± 0.03	0.06 ± 0.01[a]
Peptostreptococcaceae	0.29 ± 0.15	0.01 ± 0.01	0.29 ± 0.07	0.07 ± 0.01[a]
Rikenellaceae	0.0 ± 0.0	7.18 ± 0.72[a]	0.67 ± 0.67	4.19 ± 0.46[a]
Ruminococcaceae	4.03 ± 0.57	2.89 ± 0.39	5.76 ± 0.96	6.04 ± 0.51
S24-7	0.02 ± 0.0	33.76 ± 1.99[a]	2.68 ± 2.67	9.03 ± 0.97[a]
**Order**				
Order Clostridiales	32.29 ± 3.22	24.95 ± 1.58	40.66 ± 2.57	50.13 ± 1.43[a]
Order RF39	0.71 ± 0.13	0.63 ± 0.11	0.43 ± 0.13	0.24 ± 0.03

Each value represents the mean ± SEM of data generated from 8 to 10 mice per group. Significant differences between groups are shown for an FDR <0.05: a = significant difference between AL and CR mice. ND = Not detected.

Supplemenatry Table 4. Microbiome altered by caloric restriction (CR) in both C57BL/6JN and B6D2F1 mice.

Microbiota	Cecum		Colon	
	C57BL/6JN	B6D2F1	C57BL/6JN	B6D2F1
**Species**				
Mucispirillum schaedleri	↓	NC	↓	NC
Ruminococcus gnavus	↓	NC	NC	NC
*Bacteroides acidifaciens**	↑	↑	↑	NC
**Genus**				
*Bacteroides**	↑	↑	↑	↑
Coprococcus	NC	NC	↑	NC
Dehalobacterium	NC	↓	↑	NC
Lactobacillus	NC	↑	NC	NC
Oscillospira	↓	↓	↓	NC
Parabacteroides	↓	↓	↓	↑
Ruminococcus	↓	↓	↓	↓
Turicibacter	NC	NC	NC	NC
rc4-4	NC	↓	↑	↓
**Family**				
Erysipelotrichaceae	NC	↓	NC	↓
Lachnospiraceae	NC	↓	NC	NC
Ruminococcaceae	NC	NC	NC	NC
*S24-7**	↑	↑	↑	↑
Mogibacteriaceae	↓	↑	ND	↑
*F16**	↑	↑	↑	↑
*Rikenellaceae**	↑	↑	↑	↑
**Order**				
Clostridiales	NC	NC	↓	↑
RF39	↓	NC	NC	NC

The data are taken from Tables 1S, 2S, and 3S in the supplement. For the C57BL/6JN mice: ↑indicates microbes that showed a decrease in abundance with age but was significantly increased by CR in old mice and ↓ indicates microbes that increased in abundance with age and was significantly reduced by CR in old mice. In B6D2F1 mice: ↑indicates microbes that showed significant increase in abundance by CR in old mice and ↓ indicates microbes that showed significant decrease in abundance by CR in old mice. NC = No Significant Change. Microbes with * were found unchanged with age in C57BL/6JN mice but were altered by CR in both C57BL/6JN and B6D2F1 old mice.
