# Supplementary material for: Calorie restriction prevents age-related changes in the intestinal microbiota
Source: Aging (Albany NY). 2021 Mar 10;13(5):6298–329. doi: 10.18632/aging.202753 (PMC7993711; doi:10.18632/aging.202753)
Supplement: Supplementary Table 5 [file aging-13-202753-s003.docx]

| **Supplementary Table 5. Levels of Fecal Metabolites from C57BL/6 and B6D2F1 Mice Fed *Ad Libitum* (AL) or Caloric Restriction (CR)** | | | | | | | | | | | | | | | | | | | |
| --- | --- | --- | --- | --- | --- | --- | --- | --- | --- | --- | --- | --- | --- | --- | --- | --- | --- | --- | --- |
|  | **C57BL/6** | | | | | | | | | | | |  | **B6D2F1** | | | | | |
| **Metabolites** | **Adult AL** | | | **Adult CR** | | | **Old AL** | | | **Old CR** | | |  | **Old AL** | | | **Old CR** | | |
| 12-HETE (319.2 / 179.0) | 14.53 | ± | 0.19 | 15 | ± | 0.2 | 14.43 | ± | 0.21 | 14.9 | ± | 0.19 |  | 14.89 | ± | 0.25 | 14.91 | ± | 0.17 |
| 13-HODE (295.1 / 195.0) | 22.2 | ± | 0.35 | 22.3 | ± | 0.34 | 23.29 | ± | 0.29 | 23.1 | ± | 0.14 |  | 21.42 | ± | 0.27 | 21.23 | ± | 0.54 |
| 2-Hydroxyglutarate (147.0 / 129.0) | 20.88 | ± | 0.16 | 21.1 | ± | 0.28 | 21.13 | ± | 0.28 | 20.6 | ± | 0.08 |  | 21.84 | ± | 0.24 | 21.75 | ± | 0.46 |
| 2-Hydroxyisovaleric Acid (117.0 / 71.0) | 21.65 | ± | 0.28 | 22.1 | ± | 0.33 | 22.36 | ± | 0.73 | 22.5 | ± | 0.15 |  | 20.62 | ± | 0.37 | 21.36 | ± | 0.21 |
| 3HBA (103.0 / 59.0) | 19.32 | ± | 0.11 | 20.1 | ± | 0.2 | 18.98 | ± | 0.23a | 19.1 | ± | 0.26 |  | 19.55 | ± | 0.33 | 19.60 | ± | 0.41 |
| 3-Hydroxykynurenine (225.1 / 208.1) | 17.74 | ± | 0.29 | 18 | ± | 0.38 | 18.22 | ± | 0.13 | 18.7 | ± | 0.12 |  | 17.76 | ± | 0.16 | 18.38 | ± | 0.22 |
| 4-Hydroxybutyrate (105.0 / 77.0) | 21.56 | ± | 0.22 | 22.4 | ± | 0.15 | 22.15 | ± | 0.39 | 22.4 | ± | 0.29 |  | 22.34 | ± | 0.42 | 21.88 | ± | 0.38 |
| 4-Pyridoxic acid (182.1 / 138.0) | 22.55 | ± | 0.2 | 22.9 | ± | 0.21 | 22.44 | ± | 0.26 | 22.6 | ± | 0.15 |  | 22.31 | ± | 0.46 | 22.87 | ± | 0.22 |
| 5-Aminovaleric Acid (118.0 / 101.0) | 20.78 | ± | 0.5 | 20.5 | ± | 0.86 | 20.41 | ± | 0.64 | 19.2 | ± | 0.51 |  | 19.93 | ± | 0.79 | 19.06 | ± | 0.58 |
| Acetylcarnitine (204.1 / 85.0) | 17.88 | ± | 0.58 | 16.5 | ± | 0.69 | 17.37 | ± | 0.56 | 17.8 | ± | 0.29 |  | 17.13 | ± | 0.25 | 17.06 | ± | 0.88 |
| Adenine (134.0 / 107.0) | 24.36 | ± | 0.51 | 26.1 | ± | 0.35 | 21.68 | ± | 0.37a | 23.4 | ± | 0.61b |  | 22.17 | ± | 0.29 | 25.46 | ± | 0.25b |
| Adenosine (268.2 / 136.1) | 21.72 | ± | 0.18 | 21.5 | ± | 0.32 | 18.07 | ± | 0.31a | 20.2 | ± | 0.47b |  | 20.77 | ± | 0.19 | 20.21 | ± | 0.55 |
| Adipic Acid (144.9 / 83.0) | 16.32 | ± | 0.21 | 16.9 | ± | 0.33 | 16.34 | ± | 0.1 | 16.5 | ± | 0.15 |  | 16.17 | ± | 0.24 | 15.92 | ± | 0.26 |
| Alanine (90.0 / 44.0 (2)) | 20.29 | ± | 0.2 | 20.3 | ± | 0.23 | 21.01 | ± | 0.18a | 20.5 | ± | 0.22 |  | 21.85 | ± | 0.04 | 20.54 | ± | 0.11b |
| Allantoin (157.0 / 114.0) | 17.07 | ± | 0.26 | 16.2 | ± | 0.26 | 16.88 | ± | 0.26 | 16.5 | ± | 0.21 |  | 17.13 | ± | 0.19 | 16.40 | ± | 0.21b |
| Alpha-Ketoglutaric Acid (145.0 / 101.0) | 21.01 | ± | 0.24 | 21.6 | ± | 0.23 | 21.17 | ± | 0.32 | 21 | ± | 0.13 |  | 22.92 | ± | 0.25 | 22.48 | ± | 0.68 |
| AMP (346.1 / 79.0) | 17.08 | ± | 0.53 | 17 | ± | 0.38 | 16.83 | ± | 0.27 | 15.9 | ± | 0.31 |  | 16.20 | ± | 0.52 | 17.34 | ± | 0.58b |
| Arachidonate (303.3 / 59.0) | 22.17 | ± | 0.38 | 23.9 | ± | 0.14 | 22.84 | ± | 0.22 | 23.4 | ± | 0.07 |  | 22.64 | ± | 0.19 | 22.63 | ± | 0.24 |
| Arginine (175.0 / 70.0) | 23.53 | ± | 0.19 | 21.7 | ± | 0.26 | 25.04 | ± | 0.14a | 23.3 | ± | 0.23b |  | 25.56 | ± | 0.15 | 22.93 | ± | 0.24b |
| Asparagine (133.0 / 74.0) | 17.58 | ± | 0.38 | 15.4 | ± | 0.35 | 19.25 | ± | 0.14a | 16.4 | ± | 0.24b |  | 20.34 | ± | 0.06 | 17.36 | ± | 0.34b |
| Aspartic Acid (134.0 / 74.0) | 20.02 | ± | 0.33 | 20.8 | ± | 0.28 | 20.84 | ± | 0.28a | 19.4 | ± | 0.24b |  | 21.91 | ± | 0.13 | 20.67 | ± | 0.14b |
| Azelaic Acid (187.0 / 125.0) | 24.35 | ± | 0.32 | 26.3 | ± | 0.33 | 24.93 | ± | 0.37 | 25.8 | ± | 0.27b |  | 23.71 | ± | 0.12 | 23.24 | ± | 0.37 |
| Betaine (118.0 / 58.0) | 22.24 | ± | 0.32 | 21.9 | ± | 0.19 | 22.21 | ± | 0.36 | 21.6 | ± | 0.13 |  | 21.52 | ± | 0.23 | 21.90 | ± | 0.18 |
| Cadaverine (103.0 / 86.0) | 15.86 | ± | 0.21 | 16.1 | ± | 0.3 | 15.73 | ± | 0.08 | 15.5 | ± | 0.18 |  | 15.96 | ± | 0.09 | 15.73 | ± | 0.16 |
| Carnitine (162.0 / 85.0) | 20.54 | ± | 0.3 | 19.3 | ± | 0.25 | 19.53 | ± | 0.17a | 19.6 | ± | 0.17 |  | 20.92 | ± | 0.17 | 20.27 | ± | 0.32b |
| cGMP (344.0 / 150.0) | 18.21 | ± | 0.41 | 17.5 | ± | 0.31 | 16.74 | ± | 0.52 | 17.1 | ± | 0.46 |  | 18.64 | ± | 0.65 | 18.00 | ± | 0.79 |
| Choline (104.0 / 60.0) | 24.16 | ± | 0.21 | 24.5 | ± | 0.22 | 24.1 | ± | 0.13 | 24.6 | ± | 0.18 |  | 24.59 | ± | 0.11 | 24.31 | ± | 0.15 |
| Citraconic Acid (129.0 / 85.0) | 20.57 | ± | 0.26 | 21.1 | ± | 0.15 | 20.36 | ± | 0.11 | 20.9 | ± | 0.15 |  | 21.29 | ± | 0.35 | 20.69 | ± | 0.17 |
| Citric Acid (191.0 / 111.0) | 18.71 | ± | 0.24 | 19.2 | ± | 0.32 | 19.08 | ± | 0.33 | 19.1 | ± | 0.17 |  | 19.20 | ± | 0.19 | 18.88 | ± | 0.22 |
| Citrulline (174.0 / 131.0) | 22.88 | ± | 0.4 | 24 | ± | 0.3 | 24.06 | ± | 0.46a | 23.6 | ± | 0.47 |  | 24.82 | ± | 0.27 | 23.99 | ± | 0.19b |
| Creatine (132.0 / 90.0) | 23.03 | ± | 0.11 | 21.2 | ± | 0.51 | 22.15 | ± | 0.26 | 22.5 | ± | 0.18 |  | 22.79 | ± | 0.24 | 22.54 | ± | 0.25 |
| Creatinine (114.0 / 44.0) | 20.54 | ± | 0.68 | 17.2 | ± | 0.22 | 18.24 | ± | 0.26a | 20 | ± | 1.09 |  | 18.11 | ± | 0.23 | 17.27 | ± | 0.36 |
| Cytidine (244.2 / 112.1) | 20.81 | ± | 0.18 | 21.2 | ± | 0.15 | 19.65 | ± | 0.26a | 20.3 | ± | 0.14b |  | 21.37 | ± | 0.37 | 20.74 | ± | 0.36 |
| Cytosine (112.0 / 95.0) | 17.52 | ± | 0.66 | 20.3 | ± | 0.44 | 18.09 | ± | 0.62 | 20.1 | ± | 0.74b |  | 17.29 | ± | 0.25 | 21.24 | ± | 0.48b |
| Deoxycarnitine (147.0 / 87.0) | 17.88 | ± | 0.42 | 17.5 | ± | 0.6 | 17.44 | ± | 0.47 | 16.9 | ± | 0.38 |  | 19.81 | ± | 0.21 | 17.89 | ± | 0.44b |
| D-Leucic Acid (131.0 / 85.0) | 21.65 | ± | 0.28 | 22.1 | ± | 0.2 | 22.43 | ± | 0.5a | 22.9 | ± | 0.32 |  | 20.51 | ± | 0.17 | 22.08 | ± | 0.21b |
| Fructose (179.0 / 89.0 (3)) | 18.67 | ± | 0.84 | 17.7 | ± | 0.33 | 18.44 | ± | 0.36 | 18.1 | ± | 0.75 |  | 17.65 | ± | 0.35 | 18.29 | ± | 0.28 |
| Glucoronate (193.0 / 73.0) | 19.77 | ± | 0.5 | 19.6 | ± | 0.18 | 19.13 | ± | 0.32 | 20 | ± | 0.3b |  | 19.39 | ± | 0.28 | 19.64 | ± | 0.26 |
| Glucosamine (180.1 / 162.0) | 16.85 | ± | 0.12 | 17.6 | ± | 0.11 | 16.68 | ± | 0.2 | 16.9 | ± | 0.4 |  | 17.49 | ± | 0.24 | 17.51 | ± | 0.42 |
| Glucose (179.0 / 89.0) | 22.88 | ± | 0.57 | 22.5 | ± | 0.44 | 23.03 | ± | 0.05 | 23.7 | ± | 0.46 |  | 21.99 | ± | 0.31 | 22.97 | ± | 0.16b |
| Glutamic acid (148.0 / 84.0) | 23.11 | ± | 0.22 | 23.7 | ± | 0.34 | 24.03 | ± | 0.29a | 23.1 | ± | 0.25 |  | 25.22 | ± | 0.08 | 24.12 | ± | 0.25b |
| Glutamine (147.0 / 84.0) | 20.87 | ± | 0.36 | 20.2 | ± | 0.16 | 22.2 | ± | 0.2a | 21.5 | ± | 0.27 |  | 23.16 | ± | 0.07 | 21.17 | ± | 0.18b |
| Glyceraldehyde (89.0 / 59.0) | 16.65 | ± | 0.43 | 16.7 | ± | 0.25 | 16.9 | ± | 0.15 | 17.5 | ± | 0.37 |  | 15.99 | ± | 0.25 | 16.92 | ± | 0.18b |
| Glyceraldehyde (91.0 / 65.0) | 17.56 | ± | 0.22 | 17.6 | ± | 0.1 | 18.6 | ± | 0.16a | 17.9 | ± | 0.12b |  | 19.42 | ± | 0.08 | 17.70 | ± | 0.08b |
| Glycerate (105.0 / 75.0) | 21.74 | ± | 0.26 | 20.9 | ± | 0.31 | 21 | ± | 0.17 | 20.3 | ± | 0.12b |  | 21.26 | ± | 0.15 | 21.64 | ± | 0.16 |
| Glycine (76.0 / 30.1) | 15.21 | ± | 0.3 | 15.2 | ± | 0.26 | 15.77 | ± | 0.2 | 15.6 | ± | 0.36 |  | 16.95 | ± | 0.09 | 15.67 | ± | 0.18b |
| Guanosine (284.2 / 152.1) | 19.89 | ± | 0.15 | 18.6 | ± | 0.39 | 16.93 | ± | 0.35a | 18.4 | ± | 0.7b |  | 20.51 | ± | 0.36 | 17.83 | ± | 0.58b |
| Histamine (112.0 / 95.0 (2)) | 17.12 | ± | 0.75 | 16.5 | ± | 0.53 | 16.54 | ± | 0.17 | 16.9 | ± | 0.74 |  | 18.03 | ± | 0.88 | 16.72 | ± | 0.45 |
| Histidine (156.0 / 110.0) | 21.72 | ± | 0.24 | 21.4 | ± | 0.17 | 22.46 | ± | 0.12a | 21.9 | ± | 0.2 |  | 23.56 | ± | 0.12 | 21.58 | ± | 0.17b |
| Homoserine (120.0 / 74.0) | 20.05 | ± | 0.34 | 19.9 | ± | 0.17 | 21.04 | ± | 0.19a | 20.5 | ± | 0.3 |  | 22.14 | ± | 0.06 | 20.58 | ± | 0.16b |
| Hydroxyproline (132.0 / 86.2) | 18.47 | ± | 0.15 | 18.4 | ± | 0.24 | 18.36 | ± | 0.1 | 18.8 | ± | 0.23 |  | 18.24 | ± | 0.15 | 18.71 | ± | 0.22 |
| Hypoxanthine (135.0 / 92.0) | 25.08 | ± | 0.3 | 26.2 | ± | 0.36 | 26.37 | ± | 0.28 | 26.1 | ± | 0.28 |  | 26.96 | ± | 0.21 | 26.52 | ± | 0.43 |
| Indole-3-Acetic Acid (174.0 / 130.0) | 17.1 | ± | 0.3 | 17.8 | ± | 0.24 | 16.65 | ± | 0.44 | 17.7 | ± | 0.13 |  | 15.98 | ± | 0.23 | 16.58 | ± | 0.22 |
| Inositol (179.0 / 87.0) | 19.68 | ± | 0.4 | 19.5 | ± | 0.22 | 19.43 | ± | 0.26 | 19.7 | ± | 0.15 |  | 19.55 | ± | 0.29 | 19.62 | ± | 0.26 |
| iso-Leucine (132.0 / 86.0 (2)) | 20.04 | ± | 0.35 | 19.4 | ± | 0.23 | 21.62 | ± | 0.17a | 20.3 | ± | 0.34b |  | 22.24 | ± | 0.09 | 20.16 | ± | 0.23b |
| Kynurenic Acid (188.0 / 144.0) | 20.2 | ± | 0.34 | 21.3 | ± | 0.2 | 20.2 | ± | 0.26 | 21.1 | ± | 0.26b |  | 18.92 | ± | 0.19 | 19.70 | ± | 0.2b |
| lactate (89.0 / 43.0) | 22.14 | ± | 0.41 | 21.3 | ± | 0.63 | 21.85 | ± | 0.38 | 22.8 | ± | 0.36 |  | 21.20 | ± | 0.24 | 21.59 | ± | 0.26 |
| Lactose (341.0 / 59.0) | 19.46 | ± | 0.42 | 20.1 | ± | 0.71 | 19.25 | ± | 0.22 | 20.8 | ± | 0.75 |  | 18.37 | ± | 0.07 | 19.44 | ± | 0.24 |
| Leucine (132.0 / 86.0) | 22.36 | ± | 0.26 | 21.4 | ± | 0.19 | 23.66 | ± | 0.14a | 22.6 | ± | 0.27b |  | 24.45 | ± | 0.07 | 22.47 | ± | 0.18b |
| Linoleic Acid (279.1 / 261.0) | 21.82 | ± | 0.44 | 22.5 | ± | 0.19 | 23.16 | ± | 0.17a | 23.1 | ± | 0.06 |  | 21.03 | ± | 0.47 | 22.46 | ± | 0.28b |
| Linolenic Acid (277.1 / 259.0) | 18.29 | ± | 0.46 | 18.8 | ± | 0.23 | 19.69 | ± | 0.2b | 19.7 | ± | 0.08 |  | 17.29 | ± | 0.29 | 18.83 | ± | 0.39b |
| Lysine (147.0 / 84.0 (2)) | 22.41 | ± | 0.28 | 22.8 | ± | 0.22 | 23.97 | ± | 0.24a | 22.7 | ± | 0.27b |  | 24.77 | ± | 0.07 | 22.78 | ± | 0.18b |
| Malate (133.0 / 115.0) | 22.03 | ± | 0.35 | 22.3 | ± | 0.38 | 21.82 | ± | 0.12 | 21.1 | ± | 0.21 |  | 22.12 | ± | 0.12 | 22.88 | ± | 0.61 |
| Maleic Acid (115.0 / 71.0 (2)) | 20.32 | ± | 0.3 | 21.2 | ± | 0.14 | 20.37 | ± | 0.18 | 21 | ± | 0.12 |  | 21.07 | ± | 0.14 | 20.67 | ± | 0.16 |
| Malondialdehyde (71.0 / 41.0) | 18.75 | ± | 0.55 | 18.8 | ± | 0.36 | 18.82 | ± | 0.24 | 19.6 | ± | 0.46 |  | 18.06 | ± | 0.26 | 18.98 | ± | 0.16b |
| Margaric Acid (269.1 / 251.3) | 18.85 | ± | 0.11 | 19.4 | ± | 0.28 | 19.63 | ± | 0.34 | 19.3 | ± | 0.06 |  | 18.95 | ± | 0.16 | 18.31 | ± | 0.29 |
| Methionine (150.0 / 61.0) | 18.76 | ± | 0.29 | 18.5 | ± | 0.21 | 20.25 | ± | 0.29a | 19.3 | ± | 0.31 |  | 21.47 | ± | 0.08 | 19.18 | ± | 0.21b |
| N-AcetylGlycine (116.0 / 74.0) | 16.5 | ± | 0.75 | 18.7 | ± | 0.49 | 15.96 | ± | 0.08 | 17.8 | ± | 0.62 |  | 15.55 | ± | 0.11 | 19.33 | ± | 0.19b |
| N-Acetylneuraminate (308.1 / 87.0) | 24.47 | ± | 0.44 | 24.3 | ± | 0.32 | 24.01 | ± | 0.21a | 24.5 | ± | 0.31 |  | 24.89 | ± | 0.12 | 24.79 | ± | 0.16 |
| Nicotinic Acid (122.0 / 78.0) | 23.04 | ± | 0.18 | 24.6 | ± | 0.35 | 23.64 | ± | 0.29 | 23.6 | ± | 0.18 |  | 23.97 | ± | 0.17 | 23.88 | ± | 0.34 |
| Ornithine (133.0 / 70.0) | 17.79 | ± | 0.25 | 19.1 | ± | 0.29 | 18.97 | ± | 0.17a | 18.1 | ± | 0.37b |  | 18.44 | ± | 0.12 | 18.34 | ± | 0.21 |
| Orotate (155.0 / 111.0) | 19.57 | ± | 0.67 | 19.9 | ± | 0.37 | 19.4 | ± | 0.77 | 19.8 | ± | 0.35 |  | 18.68 | ± | 0.54 | 19.78 | ± | 0.34 |
| Oxalacetate (131.0 / 113.0) | 19.94 | ± | 0.39 | 18.4 | ± | 0.1 | 21.6 | ± | 0.17a | 19.1 | ± | 0.17b |  | 22.60 | ± | 0.06 | 19.87 | ± | 0.30b |
| Pentothenate (218.1 / 88.0) | 22.14 | ± | 0.37 | 24.2 | ± | 0.43 | 22.66 | ± | 0.44 | 22.1 | ± | 0.33 |  | 22.89 | ± | 0.23 | 23.04 | ± | 0.37 |
| Phenylalanine (166.0 / 120.0) | 23.45 | ± | 0.26 | 22.7 | ± | 0.19 | 24.71 | ± | 0.17a | 23.7 | ± | 0.21b |  | 25.55 | ± | 0.08 | 23.48 | ± | 0.18b |
| Pipecolate (130.0 / 84.0) | 17.61 | ± | 0.42 | 18.9 | ± | 0.41 | 17.25 | ± | 0.41 | 16.7 | ± | 0.17 |  | 17.79 | ± | 0.18 | 18.24 | ± | 0.41 |
| PPA (163.0 / 91.0) | 17 | ± | 0.17 | 16.8 | ± | 0.23 | 17.24 | ± | 0.15 | 17.4 | ± | 0.31 |  | 18.29 | ± | 0.14 | 18.38 | ± | 0.40 |
| Proline (116.0 / 70.0) | 20.04 | ± | 0.34 | 20.8 | ± | 0.32 | 20.76 | ± | 0.21a | 20.9 | ± | 0.29b |  | 21.50 | ± | 0.24 | 20.94 | ± | 0.14 |
| Pyroglutamic Acid (130.0 / 83.9) | 16.3 | ± | 0.36 | 17.3 | ± | 0.32 | 16.24 | ± | 0.24 | 16.5 | ± | 0.21 |  | 17.04 | ± | 0.20 | 16.87 | ± | 0.25 |
| Pyruvate (87.0 / 43.0) | 17.17 | ± | 0.1 | 17.7 | ± | 0.16 | 17.4 | ± | 0.24 | 18 | ± | 0.29b |  | 18.67 | ± | 0.09 | 18.24 | ± | 0.33 |
| Quinolinic Acid (168.0 / 150.0) | 17.52 | ± | 0.28 | 18.3 | ± | 0.24 | 17.66 | ± | 0.14 | 17.7 | ± | 0.13 |  | 17.59 | ± | 0.17 | 17.50 | ± | 0.19 |
| Reduced glutathione (306.3 / 143.1) | 15.41 | ± | 0.3 | 15.2 | ± | 0.17 | 17 | ± | 0.26a | 15.9 | ± | 0.21b |  | 17.54 | ± | 0.20 | 15.10 | ± | 0.19b |
| Serine (106.0 / 60.0 (2)) | 20.11 | ± | 0.28 | 19.6 | ± | 0.11 | 21.03 | ± | 0.17a | 20.3 | ± | 0.25 |  | 22.05 | ± | 0.04 | 20.45 | ± | 0.16b |
| Sorbitol (181.0 / 89.0) | 18.3 | ± | 0.52 | 18.2 | ± | 0.3 | 18.02 | ± | 0.35 | 17.8 | ± | 0.48 |  | 17.69 | ± | 0.10 | 18.10 | ± | 0.22 |
| Succinate (117.0 / 73.0) | 22.66 | ± | 0.4 | 21.4 | ± | 0.43 | 20.93 | ± | 0.31a | 22.3 | ± | 0.34b |  | 20.25 | ± | 0.20 | 21.92 | ± | 0.21b |
| Sucrose (341.0 / 59.0 (2)) | 19.13 | ± | 0.9 | 18.8 | ± | 0.56 | 18.04 | ± | 0.16a | 18.5 | ± | 0.68 |  | 17.74 | ± | 0.22 | 17.65 | ± | 0.19 |
| Taurine (126.0 / 108.0) | 20.23 | ± | 0.68 | 18.6 | ± | 0.43 | 20.14 | ± | 0.32 | 19 | ± | 0.15b |  | 21.64 | ± | 0.25 | 21.16 | ± | 0.31 |
| Taurocholate (514.5 / 124.0) | 18.01 | ± | 0.5 | 16.7 | ± | 0.29 | 17.8 | ± | 0.44 | 17.5 | ± | 0.25 |  | 20.09 | ± | 0.58 | 18.30 | ± | 0.14b |
| Threonine (120.0 / 74.0 (2)) | 20.06 | ± | 0.33 | 19.9 | ± | 0.18 | 21.05 | ± | 0.19a | 20.5 | ± | 0.31 |  | 22.13 | ± | 0.07 | 20.56 | ± | 0.15b |
| Tryptophan (205.1 / 146.0) | 19.84 | ± | 0.32 | 19.5 | ± | 0.38 | 20.82 | ± | 0.15a | 19.9 | ± | 0.21 |  | 21.92 | ± | 0.06 | 19.74 | ± | 0.19b |
| Tyramine (138.0 / 121.0) | 21.03 | ± | 0.27 | 21.8 | ± | 0.27 | 21.04 | ± | 0.09 | 21.3 | ± | 0.08 |  | 21.21 | ± | 0.16 | 21.04 | ± | 0.22 |
| Tyrosine (182.1 / 136.0) | 20.88 | ± | 0.25 | 20.7 | ± | 0.1 | 22.12 | ± | 0.18a | 21.4 | ± | 0.18b |  | 22.99 | ± | 0.07 | 21.03 | ± | 0.12b |
| Uracil (111.0 / 42.0) | 23.54 | ± | 0.23 | 24.7 | ± | 0.31 | 23.46 | ± | 0.34 | 24 | ± | 0.44b |  | 24.04 | ± | 0.23 | 24.87 | ± | 0.27b |
| Urate (167.0 / 124.0) | 18.65 | ± | 0.86 | 19.6 | ± | 0.58 | 16.85 | ± | 0.08 | 18.5 | ± | 0.57b |  | 18.61 | ± | 0.33 | 19.23 | ± | 0.16 |
| Uridine (245.2 / 113.1) | 19.17 | ± | 0.27 | 18.7 | ± | 0.21 | 18.4 | ± | 0.2 | 18.6 | ± | 0.33 |  | 19.50 | ± | 0.34 | 18.00 | ± | 0.31b |
| Valine (118.0 / 72.0) | 18.57 | ± | 0.28 | 18.3 | ± | 0.23 | 19.86 | ± | 0.16a | 18.9 | ± | 0.27b |  | 20.75 | ± | 0.05 | 18.84 | ± | 0.17b |
| Xanthine (151.0 / 108.0) | 24.76 | ± | 0.27 | 25.2 | ± | 0.42 | 24.41 | ± | 0.44 | 25.2 | ± | 0.51b |  | 24.76 | ± | 0.24 | 26.07 | ± | 0.30b |
| Xanthurenic Acid (204.1 / 160.0) | 19.59 | ± | 0.44 | 19.6 | ± | 0.28 | 19.69 | ± | 0.24 | 18.9 | ± | 0.17b |  | 19.65 | ± | 0.23 | 19.25 | ± | 0.18 |

Each value represents the mean ± SEM of normalized and imputed abundance data of metabolites generated from 6 mice per group. Significant differences between groups are shown for an FDR <0.05: a = significant difference between adult AL and old AL, b = significant difference between old AL and old CR. The numbers in parenthesis by the metabolite names indicates Parts/million (PPM)/Multiple Reaction Monitoring (MRM).
