# Supplementary material for: Calorie restriction prevents age-related changes in the intestinal microbiota
Source: Aging (Albany NY). 2021 Mar 10;13(5):6298–329. doi: 10.18632/aging.202753 (PMC7993711; doi:10.18632/aging.202753)
Supplement: Supplementary Table 6 [file aging-13-202753-s004.pdf]

Supplemenatry Table 6. List of the top 20 transcripts that show a significant [P (Corr) <= 0.05] and 2-fold change with age or CR in C57BL/6 mice.

Ensembl ID	Entrez ID	Log FC	FC (abs)	Regulation	Gene symbol
	**Transcripts that change with age (Old AL vs Adult AL) in C57BL/6 mice**				
ENSMUSG00000079260	100504715	5.939521	61.37254	up	*Tmppe*
ENSMUSG00000020826	18126	5.692607	51.71844	up	*Nos2*
ENSMUSG00000079494	69049	4.578009	23.8846	up	*Nat8f5*
ENSMUSG00000030004	68396	4.480648	22.32593	up	*Nat8*
ENSMUSG00000044988	83428	4.337706	20.21992	up	*Ucn3*
ENSMUSG00000051262	93674	4.168231	17.97888	up	*Nat8f3*
ENSMUSG00000035394	74453	3.714172	13.12433	up	*Cfap53*
ENSMUSG00000025194	100038628	3.380169	10.41195	up	*Gm10768*
ENSMUSG00000087361	68400	3.302278	9.864719	up	*0610043K17Rik*
ENSMUSG00000031302	245537	3.136771	8.795529	up	*Nlgn3*
ENSMUSG00000002265	18616	-4.84128	28.66614	down	*Peg3*
ENSMUSG00000054423	27062	-4.61523	24.5088	down	*Cadps*
ENSMUSG00000055193	317652	-4.57431	23.82348	down	*Klk15*
ENSMUSG00000025991	227231	-4.55286	23.47186	down	*Cps1*
ENSMUSG00000031430	78789	-3.95537	15.51261	down	*Vsig1*
ENSMUSG00000055567	329178	-3.80721	13.99862	down	*Unc80*
ENSMUSG00000027674	58869	-3.75772	13.52654	down	*Pex5l*
ENSMUSG00000031292	382253	-3.7395	13.35675	down	*Cdkl5*
ENSMUSG00000074796	269356	-3.70428	13.03465	down	*Slc4a11*
ENSMUSG00000009378	240638	-3.61335	12.23844	down	*Slc16a12*
**Transcripts that change with CR (Adult CR vs Adult AL) in C57BL/6 mice**					
ENSMUSG00000020826	18126	2.707298	6.530971	up	*Nos2*
ENSMUSG00000013611	66696	2.399487	5.276156	up	*Snx31*
ENSMUSG00000031538	18791	2.322075	5.00051	up	*Plat*
ENSMUSG00000061615	319172	2.292758	4.899918	up	*Hist1h2ab*
ENSMUSG00000033676	14402	2.282655	4.865725	up	*Gabrb3*
ENSMUSG00000044988	83428	2.013193	4.036746	up	*Ucn3*
ENSMUSG00000108218	257871	1.958138	3.8856	up	*Olfr1372-ps1*
ENSMUSG00000015702	71790	1.919249	3.782262	up	*Anxa9*
ENSMUSG00000114442	100038627	1.817609	3.524965	up	*F630042J09Rik*
ENSMUSG00000051262	93674	1.812597	3.512741	up	*Nat8f3*
ENSMUSG00000074796	269356	-3.10644	8.612539	down	*Slc4a11*
ENSMUSG00000055193	317652	-2.47794	5.571014	down	*Klk15*
ENSMUSG00000043165	16939	-2.25796	4.783151	down	*Lor*
ENSMUSG00000030205	93746	-2.1144	4.330094	down	*Gprc5d*
ENSMUSG00000031430	78789	-1.92479	3.796819	down	*Vsig1*
ENSMUSG00000099003	102465626	-1.90968	3.757266	down	*Mir7035*
ENSMUSG00000022225	17228	-1.87459	3.666969	down	*Cma1*
ENSMUSG00000044083	100504221	-1.84962	3.604044	down	*Efcab8*
ENSMUSG00000064925	104433	-1.81453	3.517459	down	*Snora62*
	99169	-1.7873	3.451673	down	*AU015228*
**Transcripts that change with CR (Old CR vs Old AL) in old C57BL/6 mice**					
ENSMUSG00000027068	241452	3.636485	12.43629	up	*Dhrs9*
ENSMUSG00000007682	13371	3.547132	11.68942	up	*Dio2*

ENSMUSG00000030963	22242	3.267541	9.630031	up	*Umod*
ENSMUSG00000030954	67133	3.209645	9.251227	up	*Gp2*
ENSMUSG00000078302	15229	3.118569	8.685258	up	*Foxd1*
ENSMUSG00000074796	269356	3.05619	8.317731	up	*Slc4a11*
ENSMUSG00000025991	227231	2.695655	6.478479	up	*Cps1*
ENSMUSG00000030205	93746	2.538617	5.810316	up	*Gprc5d*
ENSMUSG00000024912	14283	2.5329	5.78734	up	*Fosl1*
ENSMUSG00000056716	432436	2.498507	5.651002	up	*Gm5420*
ENSMUSG00000079260	100504715	-4.44553	21.789	down	*Tmppe*
ENSMUSG00000020826	18126	-4.24985	19.02536	down	*Nos2*
ENSMUSG00000030483	13088	-3.38368	10.43735	down	*Cyp2b10*
ENSMUSG00000039787	99151	-3.02324	8.129922	down	*Cercam*
ENSMUSG00000027209	75823	-2.65985	6.319671	down	*Fam227b*
ENSMUSG00000038550	229599	-2.57473	5.957578	down	*Ciart*
ENSMUSG00000000489	18591	-2.528	5.767729	down	*Pdgfb*
ENSMUSG00000044988	83428	-2.51122	5.701023	down	*Ucn3*
ENSMUSG00000062017	67928	-2.49858	5.651283	down	*Abca14*
ENSMUSG00000063171	66184	-2.45845	5.496249	down	*Rps4l*

The top 20 transcripts that show a significant fold change (FC) with either age or CR in adult and old C57BL/6 mice. Log Fold Change (Log FC) between two conditions is the difference between their respective average normalized signal values. Absolute FC (FC (abs)) is computed as (sign of Log FC) ×2|log FC|.
